# Impact of diabetes group visits on patient clinical and self-reported outcomes in community health centers

**DOI:** 10.1186/s12902-022-00972-1

**Published:** 2022-03-10

**Authors:** Arshiya A. Baig, Erin M. Staab, Amanda Benitez, Sarah P. Hermans, Sandra A. Ham, Wen Wan, Amanda Campbell, Cynthia T. Schaefer, Michael T. Quinn

**Affiliations:** 1 Department of Medicine, University of Chicago, 5841 S. Maryland Ave. MC 2007, 60637 Chicago, IL USA; 2 Enlace Chicago, Chicago, IL USA; 3 Center for Health and the Social Sciences, University of Chicago, Chicago, IL USA; 4 MidWest Clinicians’ Network, East Lansing, MI USA

**Keywords:** Group visits, Diabetes, Community Health Centers

## Abstract

**Background:**

Diabetes group visits (GVs) are a promising way to deliver high quality care but have been understudied in community health centers (CHCs), across multiple sites, or with a focus on patient-centered outcomes.

**Methods:**

We trained staff and healthcare providers from six CHCs across five Midwestern states to implement a 6-month GV program at their sites. We assessed the impact of diabetes GVs on patient clinical and self-reported outcomes and processes of care compared to patients receiving usual care at these sites during the same period using a prospective controlled study design.

**Results:**

CHCs enrolled 51 adult patients with diabetes with glycosylated hemoglobin (A1C) ≥ 8% for the GV intervention and conducted chart review of 72 patients receiving usual care. We analyzed A1C at baseline, 6, and 12 months, low-density lipoproteins (LDL), blood pressure, and patient-reported outcomes. GV patients had a larger decrease in A1C from baseline to 6 months (-1.04%, 95% CI: -1.64, -0.44) and 12 months (-1.76, 95% CI: -2.44, -1.07) compared to usual care; there was no change in blood pressure or LDL. GV patients had higher odds of receiving a flu vaccination, foot exam, eye exam, and lipid panel in the past year compared to usual care but not a dental exam, urine microalbumin test, or blood pressure check. For GV patients, diabetes distress decreased, diabetes-related quality of life improved, and self-reported frequency of healthy eating and checking blood sugar increased from baseline to 6 months, but there was no change in exercise or medication adherence.

**Conclusions:**

A diabetes GV intervention improved blood glucose levels, self-care behaviors, diabetes distress, and processes of care among adults with elevated A1Cs compared to patients receiving usual care. Future studies are needed to assess the sustainability of clinical improvements and costs of the GV model in CHCs.

**Supplementary Information:**

The online version contains supplementary material available at 10.1186/s12902-022-00972-1.

## Background

Community health centers (CHCs) provide primary care to one in twelve people in the U.S. and treat a greater proportion of adult patients with type 2 diabetes than physician offices [1]. Furthermore, CHCs play a critical role in providing primary care to many vulnerable patients, including patients who are low income and from racial and ethnic minority populations in under-served areas [2–4]. Given that more than one-third of CHC patients with diabetes have poor glycemic levels, implementing innovative models of care in this setting may improve health outcomes among vulnerable patients with diabetes [5].

Group visits (GVs) are shared patient appointments that include diabetes education in a group setting and individual visits with a medical provider [6]. There is considerable evidence for diabetes self-management education as a cornerstone to disease management [7, 8]. Due to the complexity of the disease and the need for multidisciplinary care, shared patient appointments may streamline delivery of diabetes clinical care while providing education and social support to patients. GVs have been shown to improve glycemic control, diabetes self-care, and preventive care among patients with diabetes [9–16]. However, while there is robust literature on diabetes GVs, many gaps still remain.

Systematic reviews of diabetes group visits have found that there were few data on satisfaction, patient access, or other key patient-centered outcomes [13, 14];there is a lack of reporting on patients’ diabetes distress, self-efficacy, self-empowerment, and treatment adherence in diabetes group visit intervention studies [13, 14]. There is also limited literature on implementation studies that measure real-world impacts on outcomes [14]. Lastly, while some have implemented GVs in community settings, GVs have been understudied in CHCs. Several single site GV studies have been conducted in the U.S., but none have systematically implemented GVs in multiple clinical settings across a region in the U.S.[6, 8, 14].

Our study addresses these gaps through a real world implementation of diabetes group visits in health centers across the Midwest with a focus on clinical and patient reported outcomes. In partnership with six Midwestern CHCs, we trained health centers staff on GV implementation and we conducted a study to assess changes in patient outcomes and processes of care for patients enrolled in diabetes GVs compared to those receiving usual care. We assessed changes in GV patients’ clinical outcomes and processes of care (A1C, low-density lipoproteins, and blood pressure) compared to patients who received usual care over the same timeframe, and we assessed changes in patient-centered outcomes (diabetes self-care behaviors, diabetes distress, and diabetes-related quality of life) for GV patients. Our study contributes to the current GV literature through its focus on an understudied clinical setting, its multicenter design, and assessment of patient-reported outcomes.

## Methods

We conducted a prospective controlled pilot study in which CHC staff implemented a 6-month GV program at their site. The academic team from University of Chicago partnered with the Midwest Clinicians’ Network (MWCN), a member agency of CHCs across ten Midwestern states, in conducting this study. The University of Chicago Institutional Review Board approved all study procedures, and this study was registered at clinicaltrials.gov (NCT02347514).

### Health center recruitment and training

The research team sent messages over the MWCN listserv and posted information in the MWCN newsletter to recruit CHC sites. Nineteen CHCs expressed interest, fourteen were eligible, and nine submitted applications to enroll. Six CHCs with a total of seven clinic sites from five states (Illinois, Indiana, Michigan, Nebraska, and Ohio) were enrolled in the study. Three of the CHCs were rural and three urban. Each site was asked to assemble a team of three to four members which included at least one healthcare provider. Twenty-seven CHC staff and providers were trained by University of Chicago staff and MWCN. Over the 18-month study period, the teams were invited to two in-person Learning Sessions and participated in 16 monthly webinars. More details on the training have been previously described [17].

### Patient recruitment

Eligible patients had to be at least 18 years of age, English- or Spanish-speaking, with a diagnosis of type 2 diabetes and their most recent glycosylated hemoglobin (A1C) ≥ 8.0%. They had to have had at least two visits at the CHC in the past year and one visit in the past six months. Patients who were pregnant or who had an uncontrolled psychiatric problem, cognitive impairment, or a severe physical disability were excluded. Patients were excluded from participation in the study if their primary care provider (physician, physician assistant, or nurse practitioner) deemed they would not benefit from involvement. Each CHC site generated a list of eligible patients, which was then randomly ordered by the study team. Patients were contacted by CHC staff by phone and invited to participate in the GVs until fifteen patients were enrolled. CHC group visit staff also sent letters to eligible patients or recruited them in person at clinic appointments. CHC staff recorded age, gender, race/ethnicity, and preferred language for all eligible patients who were contacted, regardless of whether they enrolled. Two CHCs did not record race/ethnicity data. Trained CHC staff obtained written informed consent from all intervention participants prior to enrollment in the study.

### Group visit intervention

The CHCs were asked to hold six monthly GVs with 8–10 patients per group. GVs included an individual medical visit by a provider, diabetes education led by a staff member or a guest speaker, and a facilitator-led discussion to encourage peer support and goal setting. Sample diabetes self-management education curricula were given to the teams to use if desired. The educational material was provided by the CHC staff in English and/or Spanish if available. The CHC staff were encouraged to provide medication refills, referrals, and vaccinations, order laboratory testing, and/or complete other process of care based on the American Diabetes Association Standards of Care during the group visit. Five out of the seven teams completed all six GVs by the end of the study period; one CHC team dropped out after the first Learning Session due to their team lead retiring and one CHC team completed only one visit. The GVs at the sites lasted from 120–210 min, the median being 120 min. Patients met with a primary care provider (PCP) individually during nearly every visit (mean 6), ranging from 5–6 across sites. The patients’ visits with the PCP lasted a median of 10 min, ranging from 10–15 min. CHC teams noted that out of the six visits they had checked vitals a median of six times (range 5–6) and refilled medications a median of 3 times (range 3–6). Sixty percent of CHCs noted completing a foot exam, 80% gave flu vaccinations, 60% gave pneumonia vaccinations, and 100% gave referrals to patients over the course of the six monthly GVs. One CHC site instituted an educational text messaging program with diabetes content to support patients between monthly GVs.

### Usual care

As a comparison group, patients were randomly selected from the original list of patients who met study inclusion criteria at baseline but had not been contacted about participating in the GV intervention. We aimed for a 2:1 ratio of usual care to intervention patients at each site.

## Measures

For patients enrolled in the GV intervention, CHC staff conducted chart abstractions at baseline, after the sixth group visit, and 12 months after baseline using an online REDCap form or an encrypted, password-protected form. CHC staff completed retrospective chart abstractions for usual care patients at 12 months. Measures collected via chart abstraction included A1C (primary outcome), low density lipoprotein (LDL), blood pressure, weight, height, and healthcare utilization (number of primary care visits, hospitalizations, and emergency room visits). Staff members also extracted available demographics, including age, gender, race/ethnicity, primary language, educational attainment, income, insurance type, family history of diabetes, diabetes duration, smoking status, and diabetes-related complications. The names of medications and dosages were abstracted; however, the medication data for usual care patients was poor quality and not used for analysis.

GV patients completed a baseline survey administered by the CHC staff prior to the first GV. They also completed an exit survey after the completion of the six monthly GVs. The surveys assessed basic demographic information, such as participant age, gender, race/ethnicity, household income, educational attainment, language spoken at home, and type of health insurance. To assess current health, patients reported number of years with diabetes, self-reported complications of diabetes, comorbidities, self-reported health status, and smoking status. Patients were also asked if they had ever received diabetes education. As secondary outcomes, we measured diabetes knowledge [18], diabetes self-management using the Summary of Diabetes Self-Care Activities scale [19], diabetes self-empowerment [20], diabetes distress [21], diabetes-related quality of life, and diabetes-related social worry [22]. Patients were also asked about satisfaction with the GVs and text messaging, if relevant. Staff recorded attendance at the monthly GVs. Usual care patients did not have survey measures collected.

## Analysis

Based on the intent-to-treat principle, all participants with available follow-up measures were analyzed. We compared participant characteristics in the intervention and usual care groups using the Student’s *t*-test or Wilcoxon rank sums for continuous variables and the Pearson χ^2^ test for categorical variables at baseline. To evaluate the 37 intervention effects, we used linear mixed models (LMM) to model repeated measures over time and to test effects of time, intervention, and interaction between time and intervention. CHC was considered as a random effect in the models. We also adjusted baseline outcome and any potential confounders such as age, gender, duration of diabetes, and insurance type. We used SAS version 9.4 (SAS Institute, Cary, NC) for all analyses and *p* < 0.05 was considered significant. We calculated that we needed data for 25–35 patients in each arm in order to detect a difference in mean change in A1C between arms of 1.0% ± 1.0 with a power of 93–98% and a two-sided significance level of 5%.

## Results

### Baseline patient characteristics

Fifty-three patients were enrolled in the GV intervention from April to May 2015. Major reasons for not agreeing to be screened or not enrolling included having other commitments like work or family, distance from the clinic, or lack of interest in the program. (Figure S1) Patients who elected not to enroll were not significantly different in age, gender, race/ethnicity, or preferred language to those who elected to enroll. Two enrolled patients were excluded due to A1C < 8% at baseline, leaving 51 intervention patients in the final analyses. At 6 months, 30 out of 51 (68%) intervention patients completed the exit survey and 45 had chart review data (88%). At 12 months, 43 out of 51 (84%) intervention patients had chart review data.

Eighty-six patients were selected for chart abstraction for the usual care group (less than the planned 2:1 ratio because one CHC did not complete chart abstraction for usual care patients and two CHC sites did not have enough remaining patients who had not been contacted about the study to meet this ratio). Fourteen patients were excluded due to missing A1C or A1C < 8% at baseline, leaving 72 usual care patients in the final analyses.

The mean age of intervention patients was 55 ± 12 years and 71% of participants were female. (Table 1) Forty-three percent were African-American, 31% non-Hispanic White, 18% Hispanic, and 8% Native American. Sixty-one percent had income that was less than $25,000 per year and the more than 20% were uninsured. Mean years since diabetes diagnosis was 13 ± 9 years, 6% reported being in excellent or very good health, and 39% had received diabetes education. Baseline average A1C of intervention patients was 10.2% ± 1.7%, mean BMI was 37.4 ± 11.4, and 49% had complications of diabetes. Usual care patients differed from the intervention patients in terms of insurance coverage and having fewer years duration of diabetes.

**Table 1:** Characteristics of Participants by Study Arm (*N* = 123)

	*Group Visit* *(N* = *51)*	*Usual Care* *(N* = *72)*	*P-value*
*Demographics*			
Age (years), (mean, SD)	55.1 (11.8)	55.4 (11.6)	0.87
Female (n, %)	36 (70.6%)	47 (65.3%)	0.54
Race/ethnicity (n, %)			0.27
Non-Hispanic White	16 (31.4%)	33 (45.8%)	
Non-Hispanic Black	22 (43.1%)	24 (33.3%)	
Hispanic or Latino	9 (17.6%)	13 (18.1%)	
Native American	4 (7.8%)	2 (2.8%)	
Language (n, %)			0.51
English	44 (86.3%)	64 (88.9%)	
Spanish	7 (13.7%)	7 (9.7%)	
Educational attainment (n, %)			
8th grade or less	4 (7.8%)	-	
Some high school	7 (13.7%)	-	
High school diploma or GED	14 (27.5%)	-	-
Some college	8 (15.7%)	-	-
2-year degree	4 (7.8%)	-	-
College graduate	4 (7.8%)	-	-
Ever had diabetes education (n,%)	20 (39.2%)		
Income (n,%)			0.28
< $25,000/year	31 (60.8%)	49 (68.1%)	
≥ $25,000/year	8 (15.7%)	21 (29.2%)	
Insurance (n,%)			0.05
Medicaid	16 (31.4%)	15 (20.8%)	
Medicare	13 (25.5%)	10 (13.9%)	
Dual-eligible	2 (3.9%)	13 (18.1%)	
Private	9 (17.6%)	20 (27.8%)	
Self-Pay/No Insurance	11 (21.6%)	14 (19.4%)	
*Health measures*			
Family history of diabetes (n,%)	38 (74.5%)	38 (52.8%)	0.48
Time since diagnosis (years), (mean, SD)	13.3 (9.4)	6.3 (4.3)	< 0.0001
General health (n,%)			
Very Good/Excellent	3 (5.9%)	-	
Fair/Poor/Good	40 (78.4%)	-	
*Clinical measures*			
Glycosylated hemoglobin (A1c) (%), (mean, SD)	10.2 (1.7%)	10.2 (1.9%)	0.81
Body mass index (BMI), (mean, SD)	37.4 (11.4)	36.9 (8.6)	0.81
Weight (lbs), (mean, SD)	221.7 (63.8)	223.8 (61.2)	0.86
Low density lipoprotein (LDL) (mg/dL)(mean, SD)	113.1 (47.2)	106.2 (41.7)	0.44
Systolic blood pressure (mmHg), (mean, SD)	135.2 (21.8)	138.7 (26.9)	0.46
Systolic blood pressure ≥ 140 mmHg or diastolic blood pressure ≥ 90 mm Hg (n, %)	20 (39.2%)	29 (40.3%)	0.66
Any complications (n,%)			0.08
Yes	25 (49.0%)	24 (33.3%)	
No	26 (51.0%)	48 (66.7%)	
Complications (n,%)			
Retinopathy	8 (15.7%)	1 (1.4%)	0.003
Neuropathy	6 (11.8%)	14 (19.4%)	0.26
Amputation	2 (3.9%)	0 (0.0%)	0.09
Nephropathy	9 (17.6%)	6 (8.3%)	0.12
Heart disease	7 (13.7%)	9 (12.5%)	0.84
Stroke	1 (2.0%)	1 (1.4%)	0.80
Peripheral arterial disease	0 (0.0%)	3 (4.2%)	0.14
Dental disease	3 (5.9%)	0 (0.0%)	0.04
Heart failure	0 (0.0%)	1 (1.4%)	0.40
*Medications*			
On insulin (n, %)	26 (51.0%)	-	
On other diabetes medications (n, %)	41 (80.3%)	-	
*Healthcare utilization*			
Had an emergency room visit in the past 6 months (n, %)	11 (21.6%)	10 (13.9%)	0.10
Had a hospitalization in the past 6 months (n, %)	7 (13.7%)	10 (13.9%)	0.21
Had a primary care visit in the past 6 months (n, %)	47 (92.2%)	65 (90.3%)	0.72

### Changes in clinical outcomes at 6-month and 12-month follow-up

**Fig. 1:**
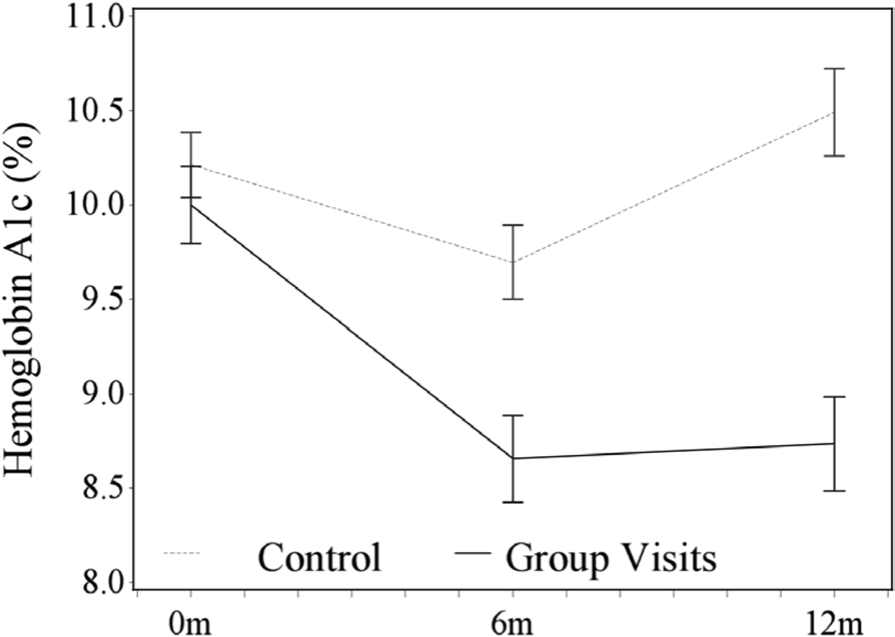
Adjusted Mean Glycosylated Hemoglobin (A1C) for Group Visit and Control Patients

Of the 51 patients enrolled, 61% attended ≥ 4 visits out of 6 group sessions. Patients attended an average of 3.5 ± 1.9 group sessions. For the primary outcome, 36 (71%) GV patients had A1C data at 6 months and 31 (61%) patients at 12 months. Of the usual care patients, 51 (71%) patients had A1C data at 6 months and 35 (49%) patients at 12 months. Figure 1 denotes patients’ average A1C by arm over time. At 6 months, the intervention and usual care patients both had significant decreases in their A1C from baseline; however, the intervention group had a larger decrease compared to the usual care group (-1.04%, (95% CI: -1.64, -0.44, *p* < 0.001). (Table 2) At 12 months, GV participants decreased their A1C from baseline (-1.26%, 95% CI: -1.79, -0.74, *p* < 0.001) and usual care patients had no significant change. At 12 months, the intervention effect was significant with a decrease in A1C of -1.76% (95% CI: -2.44, -1.07, *p* < 0.05). (Table 2) Only the intervention arm had a significant decrease in LDL from baseline to 6 months (*p* < 0.001) but this was not sustained at 12 months. The usual care arm had a significant decrease in weight compared to the intervention arm at 6-month (*p* < 0.05) and 12-month follow-up (*p* < 0.001).

**Table 2:** Changes in Clinical Outcomes by Arm over Time Compared to Baseline (Total *n* = 123)

	*Group Visit* *(n* = *51)*			*Usual Care* *(n* = *72)*		Intervention Effect^*^		
	Mean Change (95% CI)							
	6-month	12-month	6-month		12-month		6-month	12-month
Glycosylated hemoglobin (A1C, %)	-1.34 (-1.88—-0.80)†	-1.26 (-1.79—-0.74)†	-0.52 (-0.97—-0.07)‡		0.26 (-0.21 -0.76)		-1.04 (-1.64—-0.44)§	-1.76 (-2.44—-1.07)†
Low density lipoprotein (LDL) (mg/dL)	-24.1 (-36.9—-11.2)†	1.1 (-11.9 -14.1)	-6.7 (-19.5 -6.2)		12.8 (-1.6 -27.1)		-14.3 (-30.4—1.9)	-8.56 (-26.1—9.0)
Systolic blood pressure (mmHg)	-0.73 (-6.13 -4.66)	0.84 (-4.52 -6.19)	1.63 (-2.95 -6.22)		-2.00 (-6.77 -2.77)		-3.56(-10.33—3.20)	1.64 (-5.60—8.88)
Weight (lbs)	1.52 (-2.55 -5.59)	1.26 (-2.76 -5.29)	-3.20 (-6.35—-0.06)‡		-7.01 (-10.17—-3.85)†		3.97 (-1.48 -9.43)	7.52 (1.74 -13.29)†

Adjusted mean difference is from a linear mixed model that adjusts for the random effects of clinics, and repeated effects of the clinical measure over time patient. In addition, each model adjusts for the effects of study group, time, baseline clinical value, interactions group x time and baseline value x time, duration of diabetes and type of insurance. All participants with baseline and at least one follow-up measure were included to make the model robust

^*^Intervention effect was calculated by subtracting the control arm estimate from the intervention arm estimate

^†^ *p* < 0.001;

^‡^ *p* < 0.05;

^§^ *p* < 0.01

### Changes in patient-centered outcomes at 6-month follow-up for group visit patients

From baseline to 6-month follow-up, patients in the intervention group improved in days per week following a healthful eating plan, having 5 + servings of fruits and vegetables a day, and checking blood sugar. (Table 3) There were no changes in exercise, medication adherence or foot checks.

**Table 3:** Change in Patient-Reported Self-care, Knowledge, Empowerment, Distress and Diabetes-Related Quality of Life (*N* = 51)

	Baseline (*N* = 51)	6 Months (*N* = 30)	*Baseline to 6 months adjusted*	
	*Mean (SD)*	*Mean (SD)*	*Mean (CI)*	*P-value*
*Number of days in the past week*^*a*^*:*				
Followed healthful eating plan	3.26 (2.43)	4.82 (1.44)	1.70 (1.30—2.09)	< 0.0001
Ate 5 + Servings fruits and vegetables	3.82 (2.21)	4.61 (1.85)	0.73 (0.23—1.24)	0.009
Ate high fat foods	3.67 (2.22)	3.36 (2.11)	-0.28 (-0.91—0.34)	0.38
Checked feet	3.23 (2.65)	3.50 (2.39)	0.30 (-0.20—0.80)	0.24
Exercised	2.61 (2.25)	3.41 (2.58)	0.56 (0.00—1.12)	0.06
Checked blood sugar	4.77 (2.41)	6.09 (1.23)	1.34 (1.00—1.68)	< 0.0001
Took insulin injections	6.35 (1.43)	5.82 (1.74)	-0.52 (-1.18—0.14)	0.15
Took other diabetes medications	6.32 (1.45)	5.70 (1.98)	-0.49 (-1.12—0.14)	0.14
*Diabetes Knowledge*^b^ (10 is best)	6.93 (1.92)	7.50 (1.62)	0.79 (0.49—1.10)	< 0.0001
*Diabetes Empowerment*^c^ (1 = strongly disagree to 5 = strongly agree)	3.97 (0.86)	4.30 (0.53)	0.39 (0.23—0.54)	< 0.0001
*Diabetes Distress Scale*^d^ (1 = not a problem to 6 = a very serious problem)	2.36 (1.18)	1.85 (1.13)	-0.54 (-0.84—-0.24)	0.002
Emotional Burden	2.62 (1.43)	2.03 (1.41)	-0.60 (-0.98—-0.23)	0.004
Physician-related Distress	1.58 (1.21)	1.29 (0.95)	-0.43 (-0.66—-0.19)	0.002
Regimen-related Distress	2.98 (1.47)	2.04 (1.24)	-0.84 (-1.17—-0.51)	< 0.0001
Interpersonal Distress	1.86 (1.32)	1.98 (1.45)	0.01 (-0.37—0.38)	0.98
*Diabetes-related Quality of Life*^*e*^	2.25 (0.70)	1.93 (0.51)	-0.23 (-0.38—-0.08)	0.007
Dissatisfaction with glycemic stability (1 = very satisfied to 5 = very dissatisfied)	2.29 (0.81)	1.81 (0.63)	-0.34 (-0.50—-0.19)	0.0004
Social worry (1 = never to 5 = all the time)	2.17 (0.79)	2.03 (0.64)	-0.07 (-0.29—0.15)	0.55

For each measure, the adjusted mean difference is from a linear mixed model that adjusts for the random effects of patients nested within clinics, and repeated effects of the measure over time. In addition, each model adjusts for the effects of time, baseline outcomes, and interaction between baseline outcome and time. All participants who completed the baseline interview and had any follow-up data

^a^Summary of Diabetes Self-Care Activities[17]

^b^Diabetes Knowledge Questionnaire[16]

^c^Diabetes Self-empowerment Scale[18]

^d^Diabetes Distress Scale[19]

^e^Diabetes-related Quality of Life[20]

Participants improved their diabetes knowledge and diabetes self-empowerment. Participants experienced significantly less emotional burden (2.6 ± 1.4 vs. 2.0 ± 1.4 [1 = least to 6 = most distressed], *p* = 0.004), regimen distress (3.0 ± 1.5 vs. 2.0 ± 1.2, *p* = 0.014), and overall diabetes distress (2.3 ± 1.2 vs. 1.8 ± 1.1, *p* = 0.048). Diabetes-related quality of life improved overall; however, there was no change in the social worry subdomain. Dissatisfaction with diabetes control decreased significantly (2.3 ± 0.8 vs. 1.8 ± 0.6 [1 = least to 5 = most problematic], *p* = 0.029) at 6 months.

### Diabetes processes of care

Medication titration was estimable for 39 group visit patients at 6 months. Of these, 61.5% of patients increased the dosage of their diabetes medications or had a diabetes medication change from baseline to 6 months. At 12 months, 66.7% of the patients had higher dosages or changes in medications compared to 6 months. Tables S2 and S3 describe changes in diabetes processes of care from baseline to 12-month follow-up. Odds of receiving a foot exam, eye exam, yearly lipid panel, and influenza vaccine at 12 months compared to baseline were significantly higher for GV patients than usual care patients. There were no changes in receipt of a dental exam, urine microalbumin test, or blood pressure check.

### Text messaging

Of the seven patients enrolled in the text messaging program at one CHC, five completed an exit survey and all strongly or somewhat agreed that it helped them better manage their diabetes.

## Discussion

Our study found that GVs implemented in six CHCs across the Midwest led to improvements in patients’ glycemic control compared to patients receiving usual care. GV patients also demonstrated improvements in self-reported diet and home blood sugar monitoring, lower dissatisfaction with diabetes, and lower diabetes distress. Several guideline-based diabetes processes of care improved for GV patients as well compared to usual care. Some self-care behaviors, such as exercise and medication adherence, and processes of care, such as blood pressure checks and dental exams, showed no change.

In our multisite study, GV intervention patients had significant improvements in their A1C compared to patients who received usual care at the CHC sites. Previous studies testing diabetes GVs have shown improvements in blood glucose levels but few have tested these types of visits in CHC patients in underserved areas or across multiple sites [9–16]. In our study, GVs were conducted in six CHCs and 20% of study patients were uninsured and more than half were racial/ethnic minorities. While our study cannot point to the mechanisms of how GVs facilitated change in A1C, the combination of a medical visit, education, group support and provision of refills, referrals, and routine testing may have all contributed to the clinical improvements. The diabetes group visit model may need to be tested more rigorously using a randomized controlled design and with a larger patient sample in health center settings.

In terms of patient-centered outcomes, the experience of living with diabetes improved among patients who went through the GV intervention. The GV patients reported improvements in diabetes self-empowerment, diabetes distress, and diabetes-related quality of life. Previous studies have noted the association of these patient-reported outcomes to improvements in blood glucose levels outside of a group visit intervention [23]. We hypothesize that the education and group support aspects most likely contributed to improvements in patients’ self-reported outcomes. We did find that not all areas of quality of life and distress improved however; interpersonal distress and social worry about diabetes did not improve. While baseline scores were fairly low thus limiting room for improvement, this finding may point to the fact that social support from peers with diabetes can move the needle on certain aspects of living with diabetes but other more targeted interventions are needed to affect interpersonal and social worry around diabetes. Future studies are needed to understand which types of patients benefit most from GVs and explore the mechanisms by which GVs facilitate care and improve health outcomes.

Previous studies have reported that processes of care improved with diabetes GVs [24]. Our study in CHCs echoed these findings and found improvements in several guideline-based processes of care for patients in the GVs. The processes of care may have improved because the GVs provided more time for PCPs and staff to focus on preventive health and review, place referrals, and administer treatments. GVs may allow patients to receive the care they need without having to return to the center for multiple visits for refills, blood tests, and immunizations. In the era of population health management, bringing together groups of patients with poor blood glucose levels and at risk for complications of diabetes may provide efficient care that is time effective for PCPs and patients. We also found that some processes of care, such as blood pressure checks and dental exams, did not improve. These findings may have been due to high rates of assessment at baseline, such as for blood pressure checks, or, as in the case of dental exams, receipt of these services outside of the CHC which staff could not influence or perhaps facilitate.

While several areas of self-care improved, such as diet and home blood sugar monitoring, exercise and medication adherence did not. These findings may be due to high rates of medication adherence at baseline, which led to limited room for change, or, in the case of exercise, potential barriers patients had in making lifestyle change and the need for more support in increasing their physical activity. Futhermore, we found that weight decreased in the usual care group patients from baseline to 6 months and 12 months. While we cannot describe the mechanism, one potential explanation may be that CHCs offered other health programming which the usual care patients accessed since they were not occupied with the GV program or due to medication effects.

We found that there were many patients who were contacted but not interested in enrolling in the GV program. Of over 300 patients who met enrollment criteria, only 53 enrolled. Major reasons for not participating included having other commitments like work or family, distance from the clinic, or lack of interest in the program. We found no difference in patients who chose not to enroll by age, gender, race/ethnicity, or preferred language compared to those who elected to enroll. However, it is also that the improvements in A1c were due to the self-selection of patients already interested in improving their A1c agreeing to participate in the GVs. A currently ongoing RCT which we are conducting will help address this issue.

### Limitations

While our study was conducted with a sample of diverse patients across CHCs in multiple states, our study has some limitations. Our findings may not be generalizable to all CHC patients since we recruited from a select number of CHCs in the Midwest. CHCs applied to participate in the study and were therefore likely already motivated to implement GVs. Many of the intervention participants (40%) had received prior diabetes education, suggesting that they were a motivated group. We did not collect patient-reported outcomes from the usual care patients so were limited in comparing patient-reported outcomes across arms. Moreover, it is difficult to ascertain which aspects of the intervention, such as the group support, education, or medical visit may have had the strongest impact on outcomes. Thus, future research may consider comparing GVs (which include group education and individual medical visits) versus group diabetes education alone. CHC staff were trained on core components to include in their GVs but tailored their visits and the curricula to the needs of their patients and available resources, which may have contributed to heterogeneous implementation across sites. Attrition was a source of difficulty. We began the study with seven CHCs but one chose to not participate after the first learning session and one only completed one GV. Despite efforts to decrease bias by having health center staff recruit from a randomized list of eligible patients, the intervention arm had more comorbidities than the control arm at baseline. Six-month follow-up A1C data was available for 71% of all study patients and 12-month A1C data was available for 61% of intervention patients and 49% of control patients which was suboptimal. Lastly, usual care patients had shorter duration of diabetes and a different insurance mix compared to intervention patients; however, we adjusted for these differences when analyzing our clinical outcomes.

## Conclusions

Among a sample of adults with uncontrolled diabetes who receive care in CHCs, monthly diabetes GVs improved blood glucose levels, diabetes self-care, diabetes-related quality of life, and diabetes distress. Future research should assess the cost of GVs, explore the mechanisms by which GVs facilitate care, investigate which types of patients benefit most from GVs and establish best practices and training toolkits for providers and staff.

## Supplementary Information

**Additional file 1: Figure S1. **Recruitment Flowsheet. **Table S2. **Diabetes Processes of Care at Baseline (N=123). **Table S3.** Adjusted Odds Ratios for Completing Processes of Care by Group at 12 Month Follow-up Compared to Baseline (N=123).

## Data Availability

Datasets can be provided upon request.

## Abbreviations

A1C: Glycosylated hemoglobin
CHCs: Community health centers
GV: Group visits
LDL: Low density lipoprotein
LMM: Linear mixed models
MWCN: MidWest Clinicians’ Network
PCP: Primary care provider
