# Supplementary material for: Impact of diabetes group visits on patient clinical and self-reported outcomes in community health centers
Source: BMC Endocr Disord. 2022 Mar 10;22:60. doi: 10.1186/s12902-022-00972-1 (PMC8908655; doi:10.1186/s12902-022-00972-1)
Supplement: Supplementary file 1 — Additional file 1: Figure S1. Recruitment Flowsheet. Table S2. Diabetes Processes of Care at Baseline (N=123). Table S3. Adjusted Odds Ratios for Completing Processes of Care by Group at 12 Month Follow-up Compared to Baseline (N=123). [file 12902_2022_972_MOESM1_ESM.docx]

**Supplemental Material**

**Supplemental Figure S1. Recruitment Flowsheet.**

*Participants could list several reasons.

**51 patients included in final analysis. Two patients were ineligible due to baseline A1C<8%.

Not enrolled (N=91)*

- Lack of interest (N=6)
  - Don’t think they need it (N=2)
  - Don’t want to see another provider (N=1)
  - Other/unspecified (N=3)
- Time commitment/schedule conflict (N=24)
  - Work (N=8)
  - Family/caregiving (N=1)
  - Other/unspecified (N=15)
- Accessibility (N=12)
  - Transportation issues (N=2)
  - Health limitations/disability (N=3)
  - Financial burden (N=6)
  - Distance to clinic (N=1)
- Moved/out of town (N=2)
- No show/lost contact (N=9)
- Reason unknown/not documented (N=40)

Not screened (N=179)*

- Lack of interest (N=116)
  - Don’t think they need it (N=8)
  - Group setting unappealing (N=2)
  - Other/unspecified (N=106)
- Time commitment/schedule conflict (N=40)
  - Work (N=18)
  - Family/caregiving (N=9)
  - Other/unspecified (N=14)
- Accessibility (N=13)
  - Transportation issues (N=5)
  - Health limitations/disability (N=4)
  - Financial burden (N=4)
  - Distance to clinic (N=2)
- Moved/out of town (N=6)
- Lost contact/unknown (N=9)
- Type 1 diabetes (N=1)

Not contacted (N=195)

- No valid phone number (N=25)
- No response (N=99)
- Reached recruitment goal (N=71)

Enrolled

N=53**

Screened

N=144

Contacted

N=323

Eligible patients

N=518

**Supplemental Table S2. Diabetes Processes of Care at Baseline (N=123)**

|  | **Group Visit (N=51)** | **Usual Care**  **(N=72)** |  |
| --- | --- | --- | --- |
|  | **N (%)** | **N (%)** | **P-value** |
| **In the past 6 months, had:** |  |  |  |
| A1C test | 47 (92.2) | 58 (80.6) | 0.07 |
| **In the past 12 months, had:** |  |  |  |
| Blood pressure check | 50 (98.0) | 67 (93.1) | 0.21 |
| Dental exam | 10 (19.6) | 4 (5.6) | 0.02 |
| Depression screen | 38 (74.5) | 59 (81.9) | 0.32 |
| Eye exam | 13 (25.5) | 11 (15.3) | 0.16 |
| Foot exam | 18 (35.3) | 15 (20.8) | 0.07 |
| Influenza vaccine | 20 (39.2) | 24 (33.3) | 0.50 |
| Lipid panel | 34 (66.7) | 50 (69.4) | 0.74 |
| Urine microalbumin test | 36 (70.6) | 34 (47.2) | 0.01 |
| **In the past 5 years, received:** |  |  |  |
| Pneumococcal vaccine | 6 (11.8) | 4 (5.6) | 0.21 |

Supplemental Table S3. Adjusted Odds Ratios for Completing Processes of Care by Group at 12 Month Follow-up Compared to Baseline (N=123)

|  | **Group Visit** | **Usual Care** | **Intervention effect** |
| --- | --- | --- | --- |
| **Received:** | **OR (CI)** | **OR (CI)** | **OR (CI)** |
| A1C test |  |  |  |
| From baseline to 6 months | 0.53 (0.11-2.63) | 0.32 (0.13-0.74) † | 3.54 (1.00-12.5) |
| From baseline to 12 months | 0.13 (0.05-0.38) ‡ | 0.34 (0.16-0.74) † | 0.81 (0.33-1.97) |
| Annual blood pressure check | 0.94 (0.25-3.57) | 0.72 (0.26-2.01) | 0.95 (0.25-3.59) |
| Annual dental exam | 1.51 (0.24-9.33) | 1.53 (0.25-9.36) | 2.23 (0.23-21.5) |
| Annual depression screen | 0.94 (0.25-3.57) | 0.72 (0.26-2.01) | 0.95 (0.25-3.59) |
| Annual eye exam | 2.67 (0.77-9.23) | 0.44 (0.11-1.75) | 8.31 (1.69-40.8)* |
| Annual foot exam | 9.04 (2.30-35.5) † | 0.67 (0.25-1.83) | 15.5 (3.29-72.5) † |
| Annual influenza vaccine | 3.35 (0.91-12.3) | 0.66 (0.21-2.04) | 5.39 (1.05-27.7)* |
| Annual lipid panel | 2.21 (0.56-8.73) | 0.31 (0.12-0.81)* | 5.81 (1.22-27.7)* |
| Annual urine microalbumin test | 0.83 (0.25-2.75) | 0.85 (0.34-2.11) | 1.85 (0.54-6.36) |
| Pneumococcal vaccine | 4.04 (1.05-15.5)* | 10.9 (3.48-34.2) ‡ | 0.69 (0.24-2.01) |

Adjusted odds ratios are from generalized estimating equation (GEE) models that modeled each process outcome over time and tested effects of treatment, time and interaction between treatment and time. GEE models also took within-clinic associations into account and adjusted baseline process outcome and interaction between baseline process outcome and time.

*p < 0.05; † p < 0.01; ‡ p < 0.001
